# The spectrum from van der Waals to donor–acceptor bonding†

**DOI:** 10.1039/d5cp01533b

**Published:** 2025-05-27

**Authors:** Daniela Rodrigues Silva, Lucas de Azevedo Santos, Matthijs A. J. G. Koning, Célia Fonseca Guerra, Trevor A. Hamlin

**Affiliations:** a Department of Chemistry and Pharmaceutical Sciences, Amsterdam Institute of Molecular and Life Sciences (AIMMS), Vrije Universiteit Amsterdam De Boelelaan 1108 1081 HZ Amsterdam The Netherlands d.rodriguessilva@vu.nl t.a.hamlin@vu.nl

## Abstract

The chemical bond between halogenated borane Lewis acids and a variety of Lewis bases of varying strength (from strong to weak: NH_3_, MeCN, N_2_) has been quantum chemically explored using dispersion-corrected relativistic density functional theory (DFT) at ZORA-BLYP-D3(BJ)/TZ2P. We propose a unified picture of chemical bonding that exists on a continuum where weaker van der Waals (commonly referred to as “noncovalent”) interactions at longer distances transition into stronger donor–acceptor (commonly referred to as covalent) complexes at shorter distances. Remarkably, depending on the strength of the Lewis base, an intermediate regime is observed where both van der Waals and donor–acceptor complexes are observed. This study demonstrates that a covalent component is ubiquitous across the bonding spectrum, with the stability of the minima on potential energy surfaces determined by the strength of the Lewis acid–base interaction. We advocate for classifying Lewis pairs as strongly or weakly bonded based on whether their covalent interaction is strong enough to overcome the geometric penalty of bond formation. This work elucidates the fuzzy boundaries within chemical bonding.

## Abstract 2

This study demonstrates that the bonding mechanism in Lewis acid–base pairs covers a spectrum, varying in strength, not in nature, and one can shift along it by tuning the base strength or the acid rigidity.

## Graphical abstract

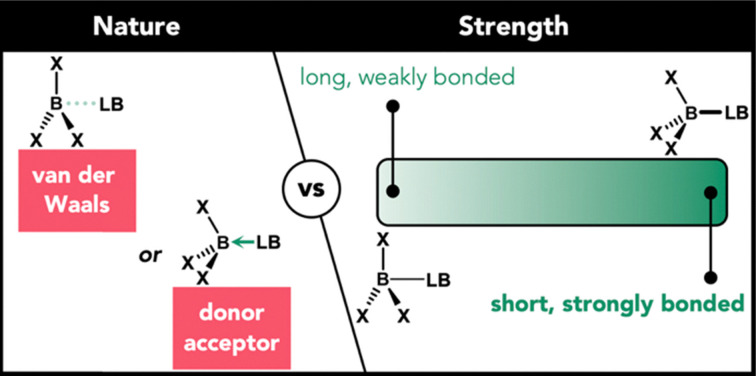

## Introduction

Chemical bonding is fundamental to all areas of chemistry as it dictates molecular structure, stability, and reactivity, influencing diverse fields from organic synthesis and catalysis to materials science and biochemistry.^1^ A deep understanding of chemical bonding enables the rational design of new molecules and materials with tailored properties,^2^ impacting advancements in drug discovery,^3^ energy storage,^4^ and nanotechnology.^5^

Covalent or donor–acceptor bonding, as defined by IUPAC, involves a region of accumulated electron density between nuclei, arising from electron sharing.^6^ Covalent bond energies typically span 40 to 150 kcal mol^−1^ for single bonds involving main block elements, with bond distances generally ranging from 0.9 to 2.0 Å, depending on the size and electronegativity of the bonded atoms.^7^ Advanced quantum chemical investigations have illuminated the nature of bonding between atoms or fragments A and B through analysis of the electronic wavefunction, providing invaluable insights into molecular structure and reactivity.^8^ A more nuanced and accurate perspective of the chemical bond beyond simple electron sharing acknowledges the synergy of multiple interactions within covalent bonds, including electrostatic attraction, Pauli repulsion, orbital interactions, and dispersion interactions.^8^

At the opposite end of the chemical bonding range, we have the intermolecular (commonly referred to as “noncovalent”) interactions that often correspond to any interaction that is weaker than covalent bonds but typically present bond strengths below 15 kcal mol^−1^. These interactions exhibit a rich diversity and encompass a broad spectrum in their own right, including ionic,^9^ hydrogen bonding,^10^ halogen bonding,^11^ chalcogen bonding,^12^ pnictogen bonding,^13^ π-interactions,^14^ and van der Waals forces (dipole–dipole interactions, dipole–induced dipole interactions, and induced dipole–induced dipole interactions),^15^ significantly expanding the chemical bonding landscape.^16^ These intermolecular interactions play a crucial role in molecular recognition and are essential for maintaining the structural integrity of biomolecules, supramolecular assemblies, and materials.^17^

Recent research has illuminated the nuanced nature of intermolecular interactions and covalent bonds, revealing that their boundaries are not always distinct.^18^ For example, it has been shown that traditional intermolecular interactions, such as hydrogen,^10*c*,10*e*,10*f*^ chalcogen,^12*c*^ and pnictogen bonding,^13^ can contain a substantial covalent character due to stabilizing donor–acceptor interactions. These strengthened interactions can exhibit bond lengths and energies comparable to traditional covalent bonds, challenging the conventional dichotomy between covalent and noncovalent bonding. Recent studies have also shown that intermolecular interactions can be strengthened to the point where they resemble covalent bonds, blurring the traditional boundaries between these two types of bonding motifs.^19^

**Scheme 1:**
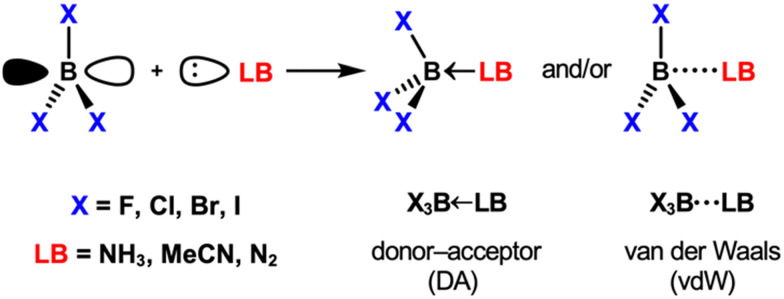
The Lewis acid–base pairs analyzed in this work.

In this work, we quantum-chemically explore the chemical bonding realm in Lewis acid–base pairs using boron trihalides BX_3_ (X_3_ = F_3_, Cl_3_, Br_3_, I_3_) as Lewis acids and Lewis bases of varying strength (from strong to weak: NH_3_, MeCN, N_2_; Scheme 1).

**Fig. 1:**
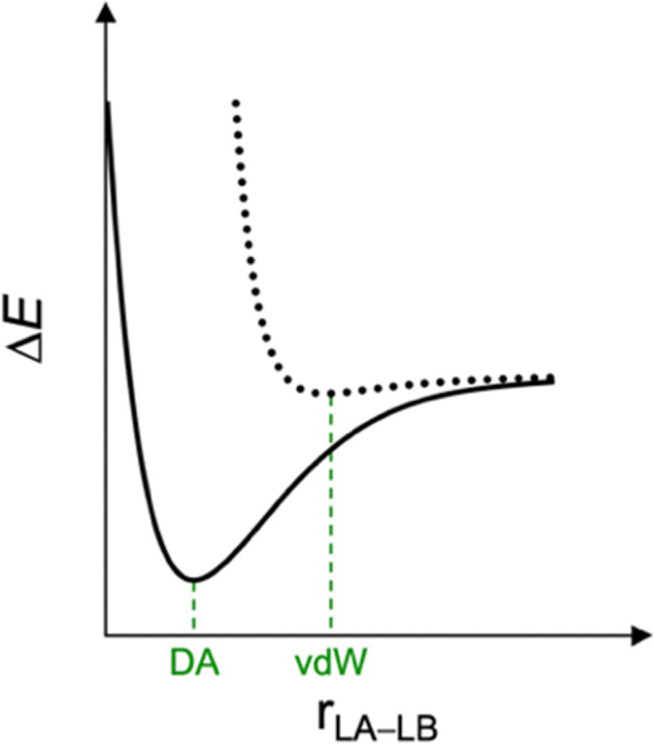
Potential energy surface for the van der Waals (vdW) and donor–acceptor (DA) bonding schemes between a Lewis acid (LA) and Lewis base (LB).

Similar to the covalent bond and intermolecular interactions dichotomy aforementioned, Lewis pairs are often classified as donor–acceptor or van der Waals depending on the stability and nature of the final adducts.^20^ Donor–acceptor complexes are classified as strong, short-bonded, and covalent in nature, whereas van der Waals complexes are weak, long-bonded, and held together by electrostatic and dispersion interactions (Fig. 1). Herein, we furnish a unified theory of a chemical bonding spectrum, which shows not only that the boundaries are fuzzy in definition but also how a bond can be tuned from one end of the spectrum to another. We advocate for the definition of a bond as strong or weak and, therefore, of Lewis pairs classified as strongly or weakly bonded. What distinguishes one Lewis pair from the other is whether the interaction between the Lewis acid and the Lewis base is strong enough to overcome the geometrical penalty inherent to the chemical bond formation. The nature of this bond is a consequence of the molecular context.

## Methods

### Computational details

All calculations were performed using the Amsterdam density functional (ADF) program (ADF2019.305).^21^ Geometries and energies were calculated at the BLYP level of the generalized gradient approximation (GGA).^22^ The DFT-D3(BJ) method developed by Grimme and coworkers was used to correct for dispersion interactions.^23^ Scalar relativistic effects were accounted for using the zeroth-order regular approximation (ZORA).^24^ Molecular orbitals (MOs) were expanded using a large, uncontracted set of Slater-type orbitals (STO): TZ2P.^25^ The TZ2P basis set is of triple-*ζ* quality, augmented by two sets of polarization functions. All electrons were treated variationally. The accuracies of the fit scheme (Zlm fit)^26^ and the integration grid (Becke grid)^27^ were set to VERY GOOD. All Lewis pairs X_3_B–LB (X_3_ = F_3_, Cl_3_, Br_3_, I_3_; LB = NH_3_, MeCN, N_2_) were optimized with both *C*_1_ (*i.e.*, without) and *C*_3v_ symmetry constraints. All optimized structures were inspected to be true minima through vibrational analyses (no imaginary frequencies).^28^ Only for the long-bonded adducts I_3_B–LB (LB = MeCN, N_2_) in *C*_3v_ symmetry we found two small imaginary frequencies (*ca.* −10 cm^−1^) because the adducts are not entirely linear along the B–N–Y (Y = C and N, respectively) bond axis. Additionally, except for X_3_B–NCMe (X = Br, I), in which MeCN approaches the Lewis acid laterally by the C≡N bond, the geometrical and energetic differences between the *C*_1_ and *C*_3v_ structures are minimal (less than 0.02 Å and 0.1 kcal mol^−1^). We have therefore used the Lewis pairs with *C*_3v_ symmetry throughout this work, as it allows us to decompose the orbital interaction into its irreducible representation contributions and a more equitable comparison between all studied adducts (the Cartesian coordinates of all studied species are provided in the ESI†). The molecular structures were illustrated using CYLview.^29^

### Activation strain and energy decomposition analyses

Insight into the nature of Lewis acid–base pairs is obtained by applying the activation strain model (ASM)^30^ in conjunction with quantitative Kohn–Sham molecular orbital (KS-MO)^31^ theory and a matching energy decomposition analysis (EDA)^32^ along the X_3_B–LB bond formation. The potential energy surface along the B–N bond formation was scanned in a stepwise manner from 1.00 to 4.00 Å with 0.005 Å per step, resulting in 121 steps in total. The overall bond energy Δ*E* is then decomposed into the respective total strain and interaction energy, Δ*E*_strain_ and Δ*E*_int_, and these values are projected onto the B–N bond distance [eqn (1)]:

1 Δ*E* = Δ*E*_strain_ + Δ*E*_int_

The strain energy Δ*E*_strain_ is the amount of energy required to deform the Lewis acid BX_3_ and Lewis base LB from their equilibrium structure to the geometry that they acquire in the final complex. Whereas the interaction energy Δ*E*_int_ corresponds to the actual energy change when the geometrically deformed BX_3_ and LB fragments are combined to form X_3_B–LB.

We further analyze the interaction energy Δ*E*_int_ within the framework of the Kohn–Sham molecular orbital (KS-MO)^30^ model by dissecting it using our canonical energy decomposition analysis (EDA)^32^ scheme into electrostatic interactions, Pauli repulsion, (attractive) orbital interactions, and dispersion corrections [eqn (2)]:

2 Δ*E*_int_ = Δ*V*_elstat_ + Δ*E*_Pauli_ + Δ*E*_oi_ + Δ*E*_disp_

The electrostatic energy Δ*V*_elstat_ corresponds to the electrostatic interactions between the unperturbed charge distributions of the fragments BX_3_ and LB, which is usually attractive. The Pauli repulsion Δ*E*_Pauli_ comprises the destabilizing interactions between occupied orbitals on either fragment (more precisely, between same-spin occupied spin-orbitals on either fragment) and arises from the antisymmetrization of the Hartree wavefunction due to the Pauli principle. The orbital interactions Δ*E*_oi_ term accounts for charge transfer (donor–acceptor interaction between an occupied orbital of one fragment with an empty orbital of the other fragment) and polarization (empty/occupied orbital mixing on one fragment due to the presence of another fragment). Finally, the dispersion energy Δ*E*_disp_ is added using Grimme's empirical correction.^23^ The PyFrag2019 program was used to facilitate these analyses.^33^

## Results and discussion

**Fig. 2:**
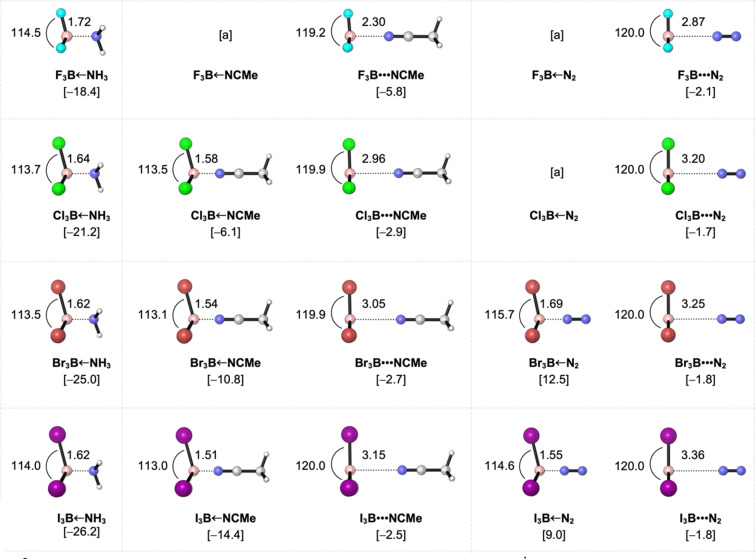
Geometries (in Å, deg.) and the electronic bond energies Δ*E* in brackets (in kcal mol^−1^) of the X_3_B–LB Lewis pairs (X_3_ = F_3_, Cl_3_, Br_3_, I_3_; LB = NH_3_, MeCN, N_2_) in *C*_3v_ symmetry, computed at ZORA-BLYP-D3(BJ)/TZ2P. ^*a*^  Lewis adduct does not exist.

The geometries and bond energies of the X_3_B–LB Lewis pairs (X_3_ = F_3_, Cl_3_, Br_3_, I_3_; LB = NH_3_, MeCN, N_2_) are analyzed and discussed. The results are summarized in Fig. 2, with detailed structural data in Table S2 of the ESI.† The strength of the B–N bond in the X_3_B–LB Lewis pairs diminishes as the Lewis base varies from NH_3_ to MeCN to N_2_ (Fig. 2). Specifically, the strong Lewis base NH_3_ forms only strongly bonded adducts with boron trihalides that go with B–N bond lengths of 1.6–1.7 Å and bonding energies (Δ*E*) ranging from −18.4 to −26.2 kcal mol^−1^. Upon forming strongly bonded adducts, the Lewis acid (BX_3_) undergoes a higher degree of pyramidalization, deviating from its trigonal planar equilibrium geometry. This structural adjustment involves decreasing the *θ*_X–B–X_ bond angle and increasing the *r*_B–X_ bond length (Table S2, ESI†). We have previously shown that the rigidity of the Lewis acids is the causal factor underlying the increase in stability of the X_3_B–NH_3_ adducts as BX_3_ is varied from BF_3_ to BI_3_.^34^ In other words, strongly bonded X_3_B–LB adducts, which characteristically have higher distortivity, become more stable as X goes from F to Cl to Br to I because the Lewis acid BX_3_ can more easily deform along this series as the corresponding B–X bond becomes weaker. This offered a new perspective on the previous rationale that ascribed the trends in Lewis acidity of boron trihalides to the strength of frontier molecular orbital interactions.^35^

In contrast, the weak Lewis base N_2_ forms only weakly bonded adducts with B–N bond lengths of 2.9–3.4 Å and Δ*E* values of −1.7 to −2.1 kcal mol^−1^. In these cases, the Lewis acid is barely deformed from its planar geometry, and the stability of the final adduct varies little and becomes somewhat less stable as BX_3_ is varied from BF_3_ to BI_3_. Note that the trends in Lewis acidity of BX_3_ towards N_2_ are exactly the opposite of those towards NH_3_ discussed above. The reversal in Lewis acidities of boron trihalides towards strong *versus* weak bases is well documented in the literature^36^ and is effectively reproduced by our DFT computations at ZORA-BLYP-D3(BJ)/TZ2P.

Lastly, the moderate Lewis base MeCN displays bond-stretch isomerism,^37^ forming both strongly and weakly bonded adducts with boron trihalides,^38^ except in the case of BF_3_, where only the long-bonded adduct is formed. The conversion of the long-bonded to the short-bonded adducts proceeds through transition states with a small barrier of *ca.* 2 kcal mol^−1^ (*i.e.*, 1.9, 1.7, and 2.0 kcal mol^−1^ for Cl_3_B–NCMe, Br_3_B–NCMe, and I_3_B–NCMe, respectively; see Table 1).

**Table 1:** Activation strain model and energy decomposition analysis terms (in kcal mol^−1^) computed at the geometries of the X_3_B–LB Lewis pairs (X_3_ = F_3_, Cl_3_, Br_3_, I_3_; LB = NH_3_, MeCN, N_2_) in *C*_3v_ symmetry^a^

X_3_B–LB	*r*_B–N_	Δ*E*	Δ*E*_strain_	Δ*E*_int_	Δ*V*_elstat_	Δ*E*_Pauli_	Δ*E*_oi_	Δ*E*_disp_
F_3_B←NH_3_	1.718	−18.4	21.1	−39.5	−88.8	123.7	−71.7	−2.7
F_3_B⋯NCMe	2.303	−5.8	2.8	−8.6	−18.0	21.3	−9.8	−2.1
F_3_B⋯N_2_	2.874	−2.1	0.1	−2.2	−2.3	3.1	−1.6	−1.3

Cl_3_B←NH_3_	1.642	−21.2	21.7	−42.9	−118.4	193.1	−112.2	−5.4
Cl_3_B←NCMe^b^	1.583	−6.1	22.1	−28.2	−101.4	193.1	−115.0	−5.0
Cl_3_B⋯NCMe^b^	2.962	−2.9	0.2	−3.1	−5.0	6.5	−1.9	−2.7
Cl_3_B⋯N_2_	3.203	−1.7	0.0	−1.7	−1.4	2.8	−1.1	−2.0

Br_3_B←NH_3_	1.622	−25.0	19.7	−44.7	−126.2	212.8	−125.2	−6.1
Br_3_B←NCMe^b^	1.540	−10.8	20.9	−31.7	−114.6	226.0	−137.3	−5.8
Br_3_B⋯NCMe^b^	3.051	−2.7	0.1	−2.8	−4.0	6.1	−1.9	−3.0
Br_3_B←N_2_^b^	1.690	12.5	12.2	0.4	−64.8	152.7	−82.6	−5.0
Br_3_B⋯N_2_^b^	3.250	−1.8	0.0	−1.8	−1.3	3.0	−1.2	−2.3

I_3_B←NH_3_	1.617	−26.2	17.0	−43.2	−132.9	231.0	−134.0	−7.2
I_3_B←NCMe^b^	1.511	−14.4	18.7	−33.1	−129.4	260.5	−157.5	−6.7
I_3_B⋯NCMe^b^	3.146	−2.5	0.0	−2.5	−3.5	6.3	−1.9	−3.5
I_3_B←N_2_^b^	1.551	9.0	14.2	−5.2	−97.9	230.2	−131.7	−5.9
I_3_B⋯N_2_^b^	3.356	−1.8	0.0	−1.8	−1.3	3.2	−1.1	−2.6

a Computed at ZORA-BLYP-D3(BJ)/TZ2P.

b The conversion barrier of the long-bonded to the short-bonded adduct is 1.9, 1.7, 14.3, 2.0, and 13.3 kcal mol^−1^ for Cl_3_B–NCMe, Br_3_B–NCMe, Br_3_B–N_2_, I_3_B–NCMe, and I_3_B–N_2_, respectively (see Table S2).

**Fig. 3:**
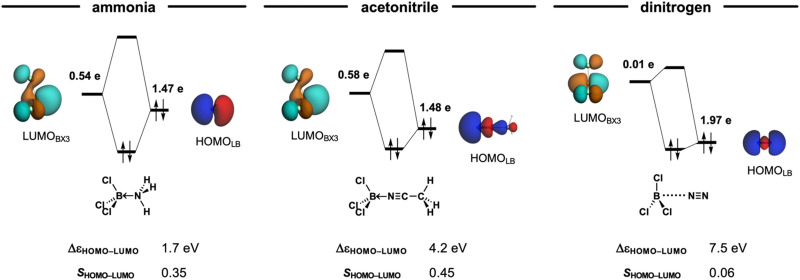
MO diagram of the HOMO_LB_–LUMO_BX3_ interaction of the Cl_3_B–LB Lewis pairs (LB = NH_3_, MeCN, N_2_) at their equilibrium geometries. Isosurface (at 0.03 au), gross Mulliken populations (in electrons), orbital energy gap (in eV), and orbital overlap of the HOMO_LB_ and LUMO_BX3_ in the a_1_ irreducible representation of the *C*_3v_ symmetry. Computed at ZORA-BLYP-D3(BJ)/TZ2P.

Classifying X_3_B–LB Lewis pairs (X_3_ = F_3_, Cl_3_, Br_3_, I_3_; LB = NH_3_, MeCN, N_2_) as either van der Waals or donor–acceptor complexes is not always straightforward. The nature of Lewis adducts is often classified by the balance of the stabilizing interactions in Δ*E*_int_ (see eqn (2) in the Methods section), namely, electrostatic stabilization Δ*V*_elstat_, orbital interactions Δ*E*_oi_, and dispersion Δ*E*_disp_. That is donor–acceptor complexes are predominantly stabilized by orbital interactions Δ*E*_oi_, whereas van der Waals complexes are mainly electrostatic Δ*V*_elstat_ and dispersion Δ*E*_disp_ in nature. However, note that, for example, the Cl_3_B←NH_3_ adduct, which is classified as a donor–acceptor complex, has a larger electrostatic than orbital interaction contribution (Δ*V*_elstat_ = −118.4 kcal mol^−1^ and Δ*E*_oi_ = −112.2 kcal mol^−1^, respectively). Similar inconsistencies can be found in Table 1. This illustrates how the classification by nature is not only limited to a few cases but also misleading, as it overlooks the fact that all complexes exhibit a covalent, that is, HOMO–LUMO molecular orbital interactions (or electrostatic or dispersion) character regardless of their stability (see Δ*E*_oi_ in Table 1 and Fig. 3).

Additionally, it is important to note that dispersion interactions (Δ*E*_disp_) are consistently weak, regardless of whether the complex is classified as van der Waals or donor–acceptor complexes in nature. The relative significance of Δ*E*_disp_ only becomes pronounced when the other stabilizing interactions, electrostatic and orbital contributions, are weakened due to the long-range separation of the interacting pair. This effect is exemplified by the Cl_3_B–NCMe adduct, where the dispersion interaction remains small, yet its relative importance increases compared to donor–acceptor interactions (Table 1) Notably, when comparing Cl_3_B←NCMe and Cl_3_B⋯NCMe, Δ*E*_oi_ varies by more than 50-fold, whereas Δ*E*_disp_ changes by only a factor of two. This highlights the secondary role of dispersion forces in determining the nature of these interactions, reinforcing the need for a more nuanced classification scheme.

**Fig. 4:**
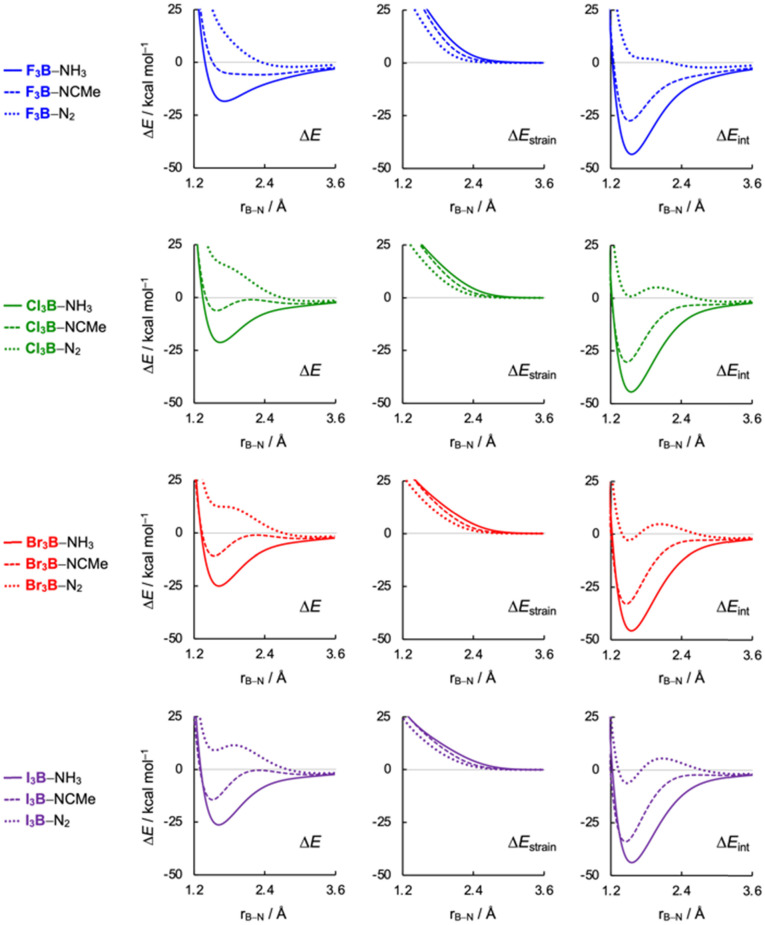
Activation strain analysis of the X_3_B–LB Lewis pairs (X_3_ = F_3_, Cl_3_, Br_3_, I_3_; LB = NH_3_, MeCN, N_2_) in *C*_3v_ symmetry projected onto the forming B–N bond distance, computed at ZORA-BLYP-D3(BJ)/TZ2P.

The key distinction among the Lewis pairs lies in the overall interaction strength, particularly whether it is sufficient to overcome the intrinsic strain energy required to bring the Lewis acid and base from their equilibrium geometries to the final complex geometry. As shown in Fig. S1 and S2 (ESI†), stronger Lewis bases exhibit more stabilizing interactions, including greater electrostatic and orbital contributions. Moreover, Pauli repulsion is also enhanced, leading to an earlier deformation of the Lewis acid (*vide infra*). The interplay between interaction energy (Δ*E*_int_) and strain energy (Δ*E*_strain_) provides critical insight into the bonding characteristics of Lewis acid–base pairs (Fig. 4). This interplay ultimately determines whether the adduct is strong or weak, shaping its overall bonding characteristics. Strong Lewis bases, such as NH_3_, enter into a stronger overall interaction, resulting in highly stable adducts because the stabilizing Δ*E*_int_ dominates over the destabilizing Δ*E*_strain_, even at shorter B–N bond distances. However, this comes at the cost of significant deformation in the Lewis acid, as NH_3_ induces an unfavorable deformation from the trigonal planar equilibrium geometry earlier along the B–N bond distance. This deformation towards a trigonal pyramidal geometry increases strain energy but is offset by the increasingly stabilizing Δ*E*_int_ (Fig. S3 and S4, ESI†), driving the system to adopt a short-bonded minimum. Notably, NH_3_ does not form a long-bonded minimum because it enters into strongly stabilizing covalent interactions at long interatomic distances with the Lewis acids (Fig. 4). In general, once the Lewis acid deforms and the interaction becomes sufficiently strong, forming the short-bonded adduct is accessible energetically.

In contrast, weak Lewis bases, such as N_2_, result in long-bonded and, therefore, weakly bonded adducts, due to extremely weak stabilizing intermolecular interactions. These complexes preserve the near-planar geometry of the Lewis acid because the weakly stabilizing Δ*E*_int_ cannot compensate for the Δ*E*_strain_ required to significantly deform the acid. As a result, the energy minimum van der Waals complex occurs at a longer bond distance where strain energy is minimally destabilizing. Importantly, N_2_ does not form short-bonded minima because its Δ*E*_int_ lacks the strength to overcome the destabilizing strain energy at shorter B–N distances. Furthermore, this general trend can be observed in I_3_B⋯N_2_, which features a shallow, short-bonded minimum in its interaction energy. This adduct highlights the nuanced balance between interaction strength and structural deformation in determining bond characteristics.

The moderate Lewis base MeCN exemplifies the intermediate regime in the chemical bonding continuum, bridging the transition from weak van der Waals interactions to strong donor–acceptor complexes. Unlike strong Lewis bases such as NH_3_, which form only short, strongly bound adducts, and weak bases like N_2_, which form only long, weakly bound complexes, MeCN uniquely exhibits both bonding motifs. Depending on the Lewis acid, it can adopt either a long-bonded minimum, stabilized primarily by electrostatic and dispersion forces, or a short-bonded minimum, where orbital interactions (charge transfer) become significant. This dual behavior underscores the continuous nature of chemical bonding, demonstrating that bonding motifs exist on a spectrum rather than as discrete categories.

Rather than representing fundamentally distinct bonding motifs, the difference between van der Waals and donor–acceptor complexes arises from the interplay between the rigidity of the Lewis acid and the strength of the Lewis base. By tuning these two factors, one can smoothly transition between these interaction types, highlighting the continuum nature of Lewis acid–base interactions and their versatility in molecular design and assembly. As discussed above, the rigidity of the Lewis acid can be modulated by selecting species with more labile bonds, which deform more readily upon bond formation. This effect is evident not only across the boron trihalide series (BX_3_) but also in heavier group-13 analogs. For example, the transition from a van der Waals complex in F_3_B–N_2_ to a donor–acceptor complex in F_3_Al–N_2_ can be attributed to the reduced rigidity of the Lewis acid as one moves from BF_3_ to AlF_3_, due to the weaker Al–F bonds^39^ (see Table S1, ESI†).

Additionally, the strain penalty can be reduced by pre-distorting the Lewis acid, as exemplified by 9-boratriptycene, which adopts a pyramidal geometry favorable for short-bonded adduct formation and can engage in a donor–acceptor interaction even with weak bases like N_2_ (Table S1, ESI†). Ultimately, the nature of a Lewis acid–base complex is not binary but reflects a spectrum of interaction strengths, governed by its equilibrium geometry. In general, more rigid Lewis acids or weaker bases tend to favor long-bonded, van der Waals interactions, whereas more flexible acids or stronger bases lead to short-bonded, donor–acceptor complexes.

## Conclusions

Using dispersion-corrected relativistic density functional theory, we have comprehensively explored the nature of Lewis acid–base interactions, providing a unified perspective on their bonding characteristics. Our findings reveal that these interactions span a continuum, encompassing both weaker van der Waals interactions at longer distances and stronger donor–acceptor complexes at shorter distances. Importantly, all bonding motifs exhibit a covalent character, with the stability of the resulting complexes determined by the balance of attractive and repulsive forces. The covalent component (Δ*E*_oi_) determines whether a complex behaves as a strong or weak Lewis pair: when both the Lewis acid and base are strong, Δ*E*_oi_ is sufficient to overcome strain, leading to a stable short-bond, donor–acceptor complex. In contrast, when the Lewis acid and base are weak, the long-bonded complex is formed where weaker forces, such as dispersion (Δ*E*_disp_), become relatively important. This study highlights the versatility of Lewis acid–base interactions in molecular assembly and offers design principles to tailor these interactions.

Delving deeper, we identify general trends in bond energy and bond length for X_3_B–LB Lewis pairs. The strength of the B–N bond varies systematically as the Lewis base transitions from NH_3_ to MeCN to N_2_. NH_3_ forms short-bonded, highly stable adducts, while N_2_ forms long-bonded, weakly interacting complexes, with MeCN exhibiting intermediate behavior where both the weaker van der Waals and donor–acceptor complexes are observed. This gradation underscores the challenges in rigidly classifying these complexes as donor–acceptor or van der Waals in nature, as they represent a continuum rather than discrete categories. This tunable and unified spectrum is modulated by the strength of the Lewis base or the rigidity of the Lewis acid, offering opportunities for precise control of interaction strength and bonding character.

Ultimately, our work underscores the continuum nature of Lewis acid–base interactions, demonstrating how subtle manipulations of acid or base properties can transition complexes between donor–acceptor and van der Waals-like bonding regimes. This emphasis on the strength rather than the nature of the classification of these interactions not only enriches our understanding of molecular assembly but also establishes a framework for the design of tailored chemical systems. These insights have far-reaching implications for advancing the field of chemical bonding and for developing innovative molecular assemblies and materials.

## Author contributions

D. R. S. and T. A. H. conceived the research idea and performed project administration. C. F. G. and T. A. H. supervised the project, and C. F. G. acquired funding. D. R. S. and M. A. J. G. K. performed the data curation, and D. R. S. and L. A. S. performed formal analysis. D. R. S., L. A. S., M. A. J. G. K., C. F. G., and T. A. H. wrote the manuscript.

## Conflicts of interest

There are no conflicts to declare.

## Supplementary Material

CP-027-D5CP01533B-s001

## Data Availability

The datasets supporting this article have been uploaded as part of the ESI.†
