# Supplementary material for: The spectrum from van der Waals to donor–acceptor bonding
Source: Phys Chem Chem Phys. 2025 May 27;27(23):12569–76. doi: 10.1039/d5cp01533b (PMC12132031; doi:10.1039/d5cp01533b)
Supplement: CP-027-D5CP01533B-s001 [file CP-027-D5CP01533B-s001.pdf]

Supplementary Information (SI) for Physical Chemistry Chemical Physics.
This journal is © the Owner Societies 2025

Supporting Information for:

The Spectrum from van der Waals to Donor–Acceptor Bonding

Daniela Rodrigues Silva,*[a] Lucas de Azevedo Santos,[a] Matthijs A.J.G. Koning,[a]

Célia Fonseca Guerra,[a] Trevor A. Hamlin*[a]

[a] *Department of Chemistry and Pharmaceutical Sciences, Amsterdam Institute of Molecular and Life Sciences (AIMMS), Vrije Universiteit Amsterdam, De Boelelaan 1108, 1081 HZ Amsterdam, The Netherlands.*
E-mail: d.rodriguessilva@vu.nl, t.a.hamlin@vu.nl

Table of contents

Figure S1. Energy decomposition analysis terms of the X_3B–LB Lewis pairs (X = F, Cl, Br, I; LB = NH_3, MeCN, N_2) projected onto the forming B–N bond distance, computed at ZORA-BLYP-D3(BJ)/TZ2P.

Figure S2. a) Schematic MO diagram of the $HOMO_{LB}$–$LUMO_{BX_3}$ interaction of the X_3B–LB Lewis pairs (X = F, Cl, Br, I; LB = NH_3, MeCN, N_2), b) isosurface (at 0.03 au) and energy (in eV) of the HOMO in the a_1 irreducible representation of the C_{3v} symmetry, c) molecular electrostatic potential (in kcal mol^{-1}, isosurface at 0.01 au) and d) VDD charges of the nitrogen atom (in milli-electrons) of the Lewis base. Computed at ZORA-BLYP-D3(BJ)/TZ2P.

Figure S3. Activation strain model terms of the I_3B–LB Lewis pairs (LB = NH_3, MeCN, N_2) projected onto the forming B–N bond distance, computed at ZORA-BLYP-D3(BJ)/TZ2P.

Figure S4. Energy decomposition analysis terms of the I_3B–LB Lewis pairs (LB = NH_3, MeCN, N_2) projected onto the forming B–N bond distance, computed at ZORA-BLYP-D3(BJ)/TZ2P.

Table S1. Activation strain model and energy decomposition analysis terms (in kcal mol^{-1}) computed at the geometries of the X_3Tr–LB Lewis pairs (TrX_3 = AlF_3, $AlCl_3$, BTri; LB = NH_3, MeCN, N_2) in C_{3v} symmetry.

Table S2. Cartesian coordinates (Å), energies (kcal mol^{-1}), and the number of imaginary vibrational frequencies (N_{imag}) of the optimized Lewis acid, Lewis bases, and X_3B–LB Lewis pairs (X = F, Cl, Br, I; LB = N H_3, MeCN, N_2) computed at ZORA-BLYP-D3(BJ)/TZ2P.

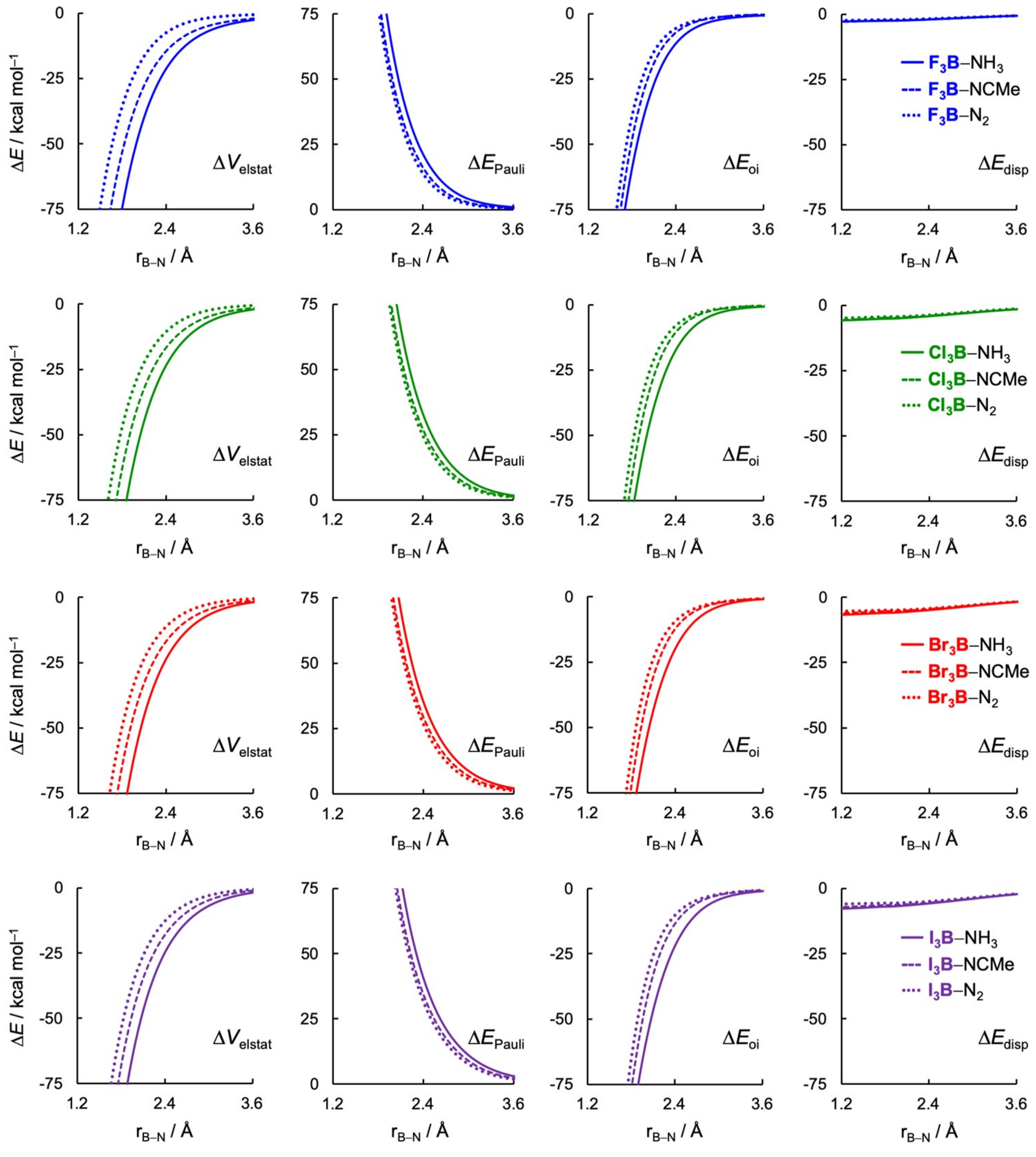

Figure S1. Energy decomposition analysis terms of the X_3B–LB Lewis pairs (X = F, Cl, Br, I; LB = NH_3, MeCN, N_2) projected onto the forming B–N bond distance, computed at ZORA-BLYP-D3(BJ)/TZ2P.

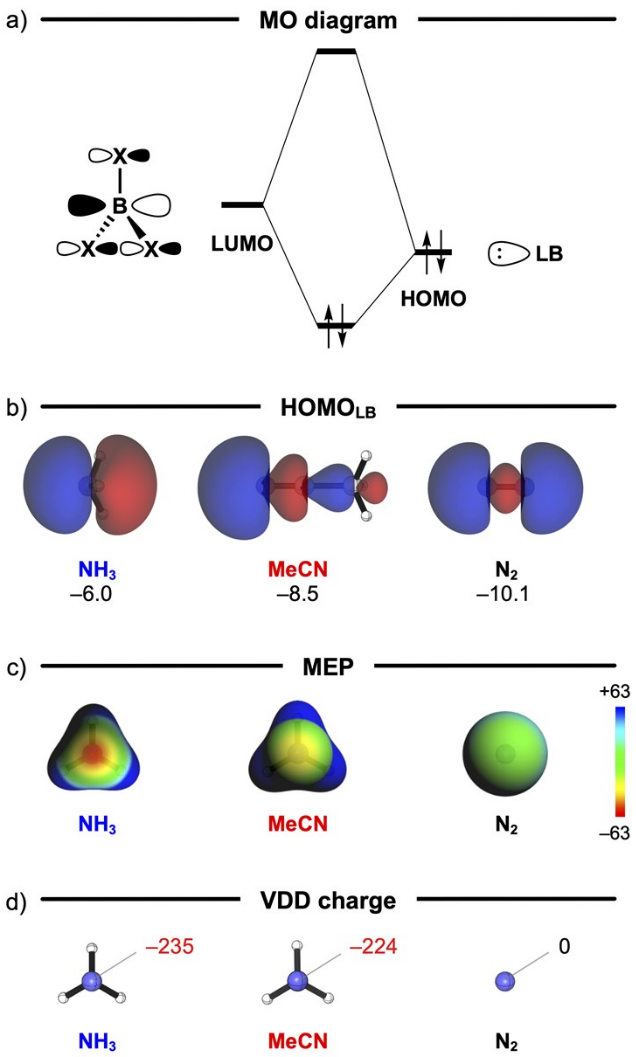

Figure S2. a) Schematic MO diagram of the HOMO$_{LB}$–LUMO$_{BX_3}$ interaction of the X_3B–LB Lewis pairs (X = F, Cl, Br, I; LB = NH_3, MeCN, N_2), b) isosurface (at 0.03 au) and energy (in eV) of the HOMO in the a_1 irreducible representation of the C_{3v} symmetry, c) molecular electrostatic potential (in kcal mol^{-1}, isosurface at 0.01 au) and d) VDD charges of the nitrogen atom (in milli-electrons) of the Lewis base. Computed at ZORA-BLYP-D3(BJ)/TZ2P.

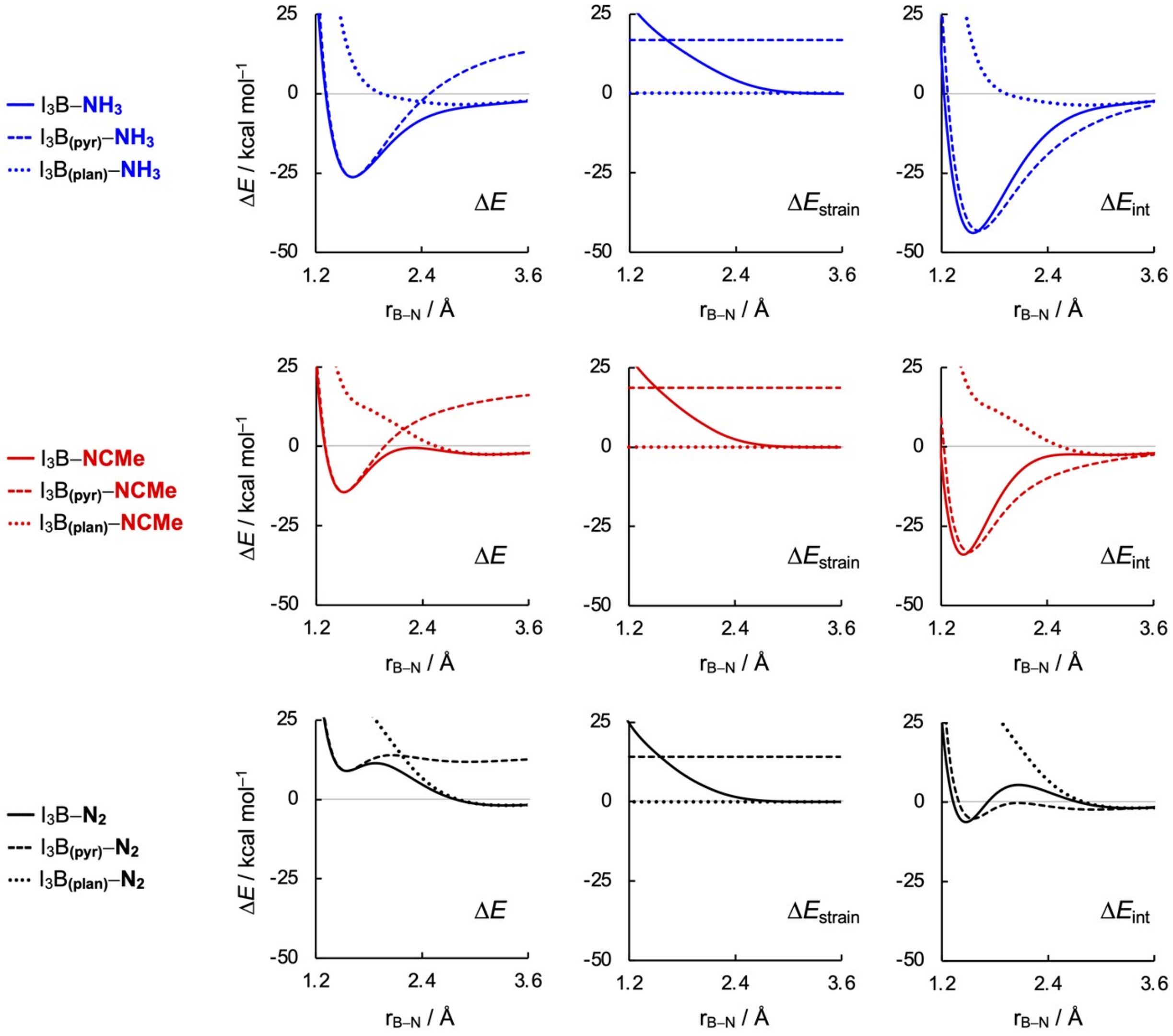

Figure S3. Activation strain model terms of the I_3B–LB Lewis pairs (LB = NH_3, MeCN, N_2) projected onto the forming B–N bond distance, computed at ZORA-BLYP-D3(BJ)/TZ2P.

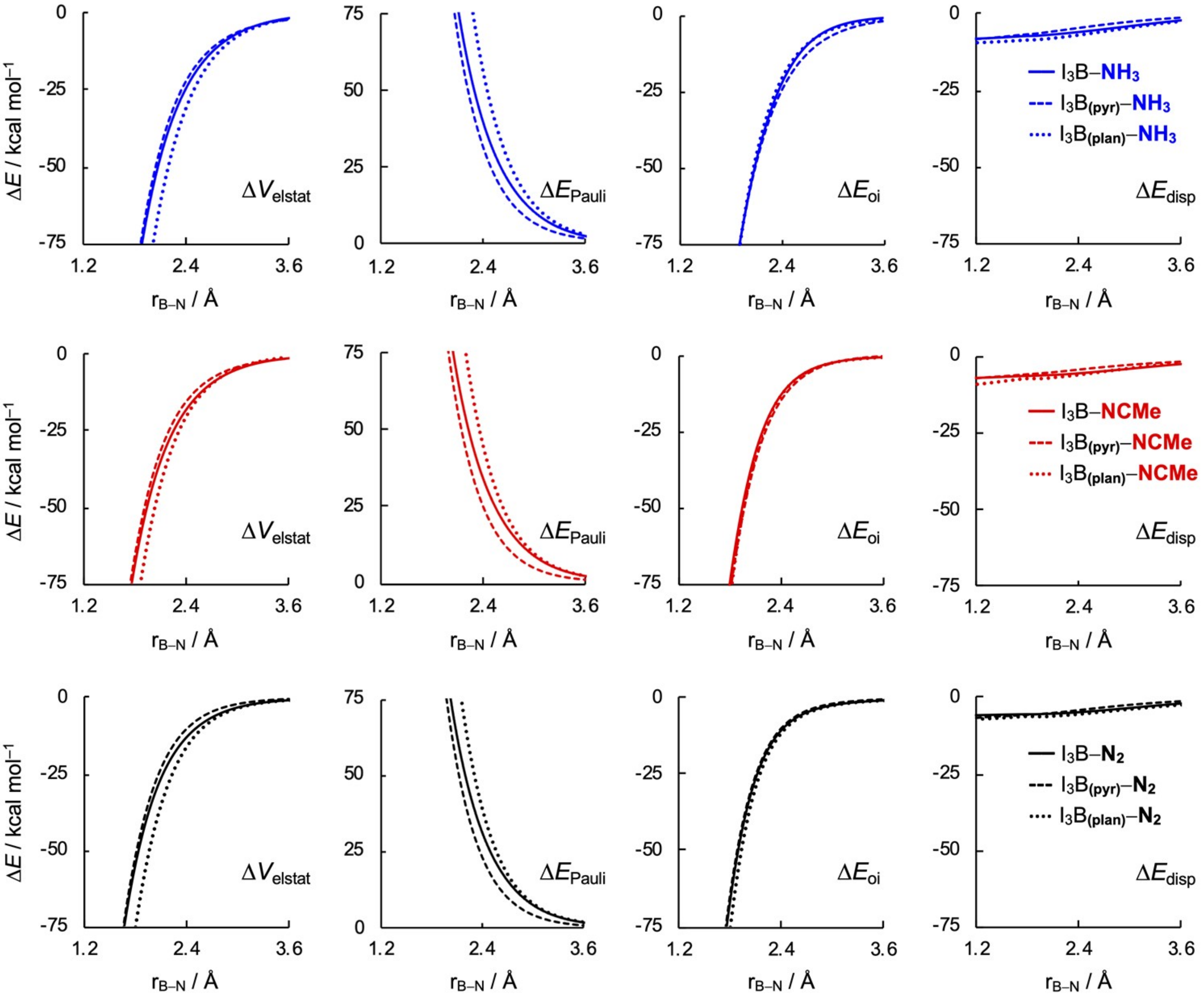

Figure S4. Energy decomposition analysis terms of the I_3B–LB Lewis pairs (LB = NH_3, MeCN, N_2) projected onto the forming B–N bond distance, computed at ZORA-BLYP-D3(BJ)/TZ2P.

Table S1. Activation strain model and energy decomposition analysis terms (in kcal mol^{-1}) computed at the geometries of the X_3Tr–LB Lewis pairs (TrX_3 = AlF_3, $AlCl_3$, BTri[b]; LB = NH_3, MeCN, N_2) in C_{3v} symmetry.[a]

X_3B–LB	$r_{Tr–N}$	ΔE	ΔE_{strain}	ΔE_{int}	ΔV_{elstat}	ΔE_{Pauli}	ΔE_{oi}	ΔE_{disp}
**$F_3Al \leftarrow NH_3$**	2.017	–38.3	6.4	–44.7	–69.0	61.6	–34.2	–3.1
**$F_3Al \leftarrow NCMe$**	1.993	–30.2	6.7	–36.9	–50.9	50.4	–33.6	–2.8
**$F_3Al \leftarrow N_2$**	2.182	–10.1	1.8	–11.9	–17.6	26.4	–18.3	–2.4
**$Cl_3Al \leftarrow NH_3$**	2.021	–35.8	6.1	–41.9	–76.7	82.3	–42.5	–5.0
**$Cl_3Al \leftarrow NCMe$**	1.985	–27.3	6.6	–33.9	–59.1	72.0	–42.2	–4.6
**$Cl_3Al \leftarrow N_2$**	2.223	–7.1	1.9	–9.0	–21.3	37.0	–20.8	–3.9
**$TriB \leftarrow N_2$[b]**	1.514	–17.4	9.2	–26.6	–64.7	144.5	–100.2	–6.2

[a] Computed at ZORA-BLYP-D3(BJ)/TZ2P; Cartesian coordinates are provided in Table S2. [b] BTrip: 9-boratriptycene.

Table S2. Cartesian coordinates (Å), energies (electronic *E* and enthalpy *H*, in kcal mol^{-1}), and the number of imaginary vibrational frequencies (N_{imag}) of the optimized BX_3 Lewis acid, LB Lewis bases, and X_3B–LB Lewis pairs (X = F, Cl, Br, I; LB = NH_3, MeCN, N_2) computed at ZORA-BLYP-D3(BJ)/TZ2P.

BF_3 (D_{3h})

E = -525.1

H = -514.8

N_{imag} = 0

B	0.00000000	0.00000000	0.00000000
F	0.66371319	1.14958496	0.00000000
F	0.66371319	-1.14958496	0.00000000
F	-1.32742637	0.00000000	0.00000000

BCl_3 (D_{3h})

E = -346.0

H = -338.1

N_{imag} = 0

B	0.00000000	0.00000000	0.00000000
Cl	0.87894830	1.52238311	0.00000000
Cl	0.87894830	-1.52238311	0.00000000
Cl	-1.75789659	0.00000000	0.00000000

BBr_3 (D_{3h})

E = -295.3

H = -288.0

N_{imag} = 0

B	0.00000000	0.00000000	0.00000000
Br	0.96111184	1.66469455	0.00000000
Br	0.96111184	-1.66469455	0.00000000
Br	-1.92222369	0.00000000	0.00000000

BI_3 (D_{3h})

E = -243.6

H = -236.7

N_{imag} = 0

B	0.00000000	0.00000000	0.00000000
I	1.07259328	1.85778606	0.00000000
I	1.07259328	-1.85778606	0.00000000
I	-2.14518657	0.00000000	0.00000000

AlF_3 (D_{3h})

E = -474.2

H = -466.1

***N**$_{imag}$* = 0

Al	0.00000000	0.00000000	0.00000000
F	0.82547289	1.42976100	0.00000000
F	0.82547289	-1.42976100	0.00000000
F	-1.65094579	-0.00000000	0.00000000

$AlCl_3$ (D_{3h})

E = -319.0

H = -312.1

***N**$_{imag}$* = 0

Al	0.00000000	0.00000000	0.00000000
Cl	1.04388600	1.80806300	0.00000000
Cl	1.04388600	-1.80806300	0.00000000
Cl	-2.08777200	0.00000000	0.00000000

***BTri* (*C_{3v}*)**

E = -4942.0

H = -4773.3

***N**$_{imag}$* = 0

B	0.00000000	0.00000000	-1.29474788
C	2.75661373	0.00000000	-1.49660892
C	3.92657070	0.00000000	-0.72001254
H	4.75808227	0.00000000	1.27134276
C	-0.75453580	1.30689435	-0.87596704
C	-1.29880871	2.24960268	1.31134763
C	-1.92432558	3.33302967	0.67473664
C	-0.71682805	1.24158261	0.54797962
H	-2.44915627	4.24206309	-1.20901089
H	-1.41588717	2.45238852	-2.58223422
C	-0.75453580	-1.30689435	-0.87596704
H	0.00000000	0.00000000	2.21364922
C	1.43365611	0.00000000	0.54797962
C	-0.71682805	-1.24158261	0.54797962
C	0.00000000	0.00000000	1.12075007
H	2.53436286	0.00000000	2.39854316
C	1.50907160	0.00000000	-0.87596704
H	-1.41588717	-2.45238852	-2.58223422
H	-2.44915627	-4.24206309	-1.20901089
C	-1.29880871	-2.24960268	1.31134763
C	-1.92432558	-3.33302967	0.67473664
C	-1.96328535	-3.40050998	-0.72001254
C	-1.37830686	-2.38729752	-1.49660892
H	-2.37904114	-4.12062012	1.27134276
H	-1.26718143	-2.19482262	2.39854316
H	2.83177434	0.00000000	-2.58223422

H	4.89831253	0.00000000	-1.20901089
C	2.59761743	0.00000000	1.31134763
C	3.84865116	0.00000000	0.67473664
C	-1.37830686	2.38729752	-1.49660892
C	-1.96328535	3.40050998	-0.72001254
H	-2.37904114	4.12062012	1.27134276
H	-1.26718143	2.19482262	2.39854316

***N_2* (*D_{lin}*)**

E = -372.6

H = -367.2

N_{imag} = 0

N	0.00000000	0.00000000	0.55139102
N	0.00000000	0.00000000	-0.55139102

***MeCN* (*C_{3v}*)**

E = -815.9

H = -785.5

N_{imag} = 0

N	0.00000000	0.00000000	2.13011733
C	0.00000000	0.00000000	0.96987558
C	0.00000000	0.00000000	-0.49116968
H	0.51423793	0.89068623	-0.86960774
H	-1.02847587	0.00000000	-0.86960774
H	0.51423793	-0.89068623	-0.86960774

***NH_3* (*C_{3v}*)**

E = -434.9

H = -411.7

N_{imag} = 0

N	0.00000000	0.00000000	-0.28896113
H	0.47327176	0.81973074	0.09632038
H	0.47327176	-0.81973074	0.09632038
H	-0.94654353	0.00000000	0.09632038

***F_3B–N_2* (*C_{3v}*)**

E = -899.8

H = -883.3

N_{imag} = 0

B	0.00000000	0.00000000	-1.17108282
F	0.66395733	-1.15000783	-1.18835391
F	-1.32791466	0.00000000	-1.18835391
F	0.66395733	1.15000783	-1.18835391
N	0.00000000	0.00000000	1.70284925

N	0.00000000	0.00000000	2.80480224

***F_3B–N_2* (*C_1*)**
E = -899.8
H = -883.3
N_{imag} = 0

B	-0.00005831	0.00000018	-1.17124584
F	0.66393717	-1.14999002	-1.18836143
F	-1.32797174	0.00000019	-1.18871303
F	0.66393715	1.14999036	-1.18836390
N	0.00003558	0.00000007	1.70311873
N	0.00012014	-0.00000079	2.80507241

***F_3B–NCMe* (*C_{3v}*)**
E = -1346.8
H = -1305.7
N_{imag} = 0

B	0.00000000	0.00000000	-2.64353783
F	0.66620444	-1.15389994	-2.76692276
F	-1.33240889	0.00000000	-2.76692276
F	0.66620444	1.15389994	-2.76692276
N	0.00000000	0.00000000	-0.34063151
C	0.00000000	0.00000000	2.27320736
C	0.00000000	0.00000000	0.81556209
H	0.51486900	-0.89177926	2.64759650
H	-1.02973799	0.00000000	2.64759650
H	0.51486900	0.89177926	2.64759650

***F_3B–NCMe* (*C_1*)**
E = -1346.8
H = -1305.1
N_{imag} = 0

B	0.20921694	0.03127362	-2.64071886
F	1.02152843	-1.02921934	-2.71373490
F	-1.09921263	-0.13234608	-2.86683431
F	0.73368000	1.26039816	-2.70458015
N	0.02826079	0.00022401	-0.33674188
C	-0.18410777	-0.02676498	2.26848665
C	-0.06570685	-0.01168272	0.81563616
H	0.26671459	-0.94046605	2.67153767
H	-1.24019059	0.00680462	2.55846064
H	0.32981709	0.84177877	2.69511030

***F_3B–NH_3* (*C_{3v}*)**

E = -978.4
H = -942.6
$\boldsymbol{N_{imag}}$ = 0

B	0.00000000	0.00000000	-0.89744610
F	0.67248909	-1.16478526	-1.22668402
F	-1.34497817	0.00000000	-1.22668402
F	0.67248909	1.16478526	-1.22668402
N	0.00000000	0.00000000	0.82030656
H	0.95958951	0.00000000	1.17435536
H	-0.47979476	-0.83102889	1.17435536
H	-0.47979476	0.83102889	1.17435536

F_3B–NH_3 (C_1)
E = -978.4
H = -942.6
$\boldsymbol{N_{imag}}$ = 0

B	-0.00001149	0.00000004	-0.89749553
F	0.67248866	-1.16479344	-1.22667802
F	-1.34498454	0.00000014	-1.22674774
F	0.67248886	1.16479331	-1.22667830
N	-0.00000844	0.00000003	0.82031572
H	0.95958846	0.00000014	1.17433109
H	-0.47978061	-0.83102130	1.17441351
H	-0.47978092	0.83102108	1.17441377

Cl_3B–N_2 (C_{3v})
E = -720.3
H = -707.3
$\boldsymbol{N_{imag}}$ = 0

B	0.00000000	0.00000000	-1.34009135
Cl	0.87878899	-1.52210719	-1.35032235
Cl	-1.75757799	0.00000000	-1.35032235
Cl	0.87878899	1.52210719	-1.35032235
N	0.00000000	0.00000000	1.86282642
N	0.00000000	0.00000000	2.96525921

Cl_3B–N_2 (C_1)
E = -720.3
H = -707.3
$\boldsymbol{N_{imag}}$ = 0

B	-0.00041571	-0.00000009	-1.34012829
Cl	0.87854460	-1.52199951	-1.34920653
Cl	-1.75801474	-0.00000002	-1.35268737
Cl	0.87854454	1.52199936	-1.34920572
N	-0.00071947	-0.00000011	1.86291293

N	0.00206079	0.00000037	2.96534221

***Cl_3B–NCMe* (*SB*) (*C_{3v}*)**
E = -1168.1
H = -1128.4
N_{imag} = 0

B	0.00000000	0.00000000	-2.15053323
Cl	0.89375518	-1.54802937	-2.63168161
Cl	-1.78751035	0.00000000	-2.63168161
Cl	0.89375518	1.54802937	-2.63168161
N	0.00000000	0.00000000	-0.56786704
C	0.00000000	0.00000000	2.03140064
C	0.00000000	0.00000000	0.58262334
H	0.51625342	-0.89417716	2.39998708
H	-1.03250685	0.00000000	2.39998708
H	0.51625342	0.89417716	2.39998708

***Cl_3B–NCMe* (*SB*) (*C_1*)**
E = -1168.1
H = -1128.4
N_{imag} = 0

B	-0.00056906	0.00000002	-2.15051879
Cl	0.89426400	-1.54796876	-2.62998294
Cl	-1.78711024	-0.00000002	-2.63501128
Cl	0.89426400	1.54796871	-2.62998316
N	-0.00259338	0.00000010	-0.56787435
C	0.00037341	-0.00000001	2.03138294
C	-0.00178193	0.00000009	0.58261432
H	0.51730240	-0.89416717	2.39907986
H	-1.03145162	-0.00000004	2.40175348
H	0.51730240	0.89416708	2.39908003

***Cl_3B–NCMe* (*TS*) (*C_{3v}*)**
E = -1162.9
H = -1124.7
N_{imag} = 1, ν = -163.1708 cm^{-1}

B	0.00000000	0.00000000	2.49381444
Cl	-0.88492512	-1.53273527	2.75150018
Cl	1.76985024	-0.00000000	2.75150018
Cl	-0.88492512	1.53273527	2.75150018
N	-0.00000000	-0.00000000	0.32913574
C	-0.00000000	-0.00000000	-2.28268658
C	-0.00000000	-0.00000000	-0.82627547
H	-0.51504312	-0.89208084	-2.65616289
H	1.03008623	-0.00000000	-2.65616289

H	-0.51504312	0.89208084	-2.65616289

***Cl_3B–NCMe* (*TS*) (*C_1*)**

E = -1162.9

H = -1124.7

N_{imag} = 1, ν = -163.3186 cm^{-1}

B	0.00009070	0.00026725	-1.61390445
Cl	0.85367878	-1.55175046	-1.86164331
Cl	-1.76919720	0.03470148	-1.87273774
Cl	0.91598646	1.51302141	-1.88014103
N	-0.00150075	0.01123418	0.55103077
C	0.00021833	-0.00180156	3.16284833
C	-0.00062974	0.00535300	1.70642423
H	0.56773015	-0.86354707	3.53159035
H	-1.02788648	-0.06501366	3.53652617
H	0.46150975	0.91753544	3.54054681

***Cl_3B–NCMe* (*LB*) (*C_{3v}*)**

E = -1164.8

H = -1127.1

N_{imag} = 0

B	0.00000000	0.00000000	-2.15896028
Cl	0.87972692	-1.52373172	-2.20777284
Cl	-1.75945384	0.00000000	-2.20777284
Cl	0.87972692	1.52373172	-2.20777284
N	0.00000000	0.00000000	0.80263466
C	0.00000000	0.00000000	3.42167755
C	0.00000000	0.00000000	1.96174363
H	0.51441901	-0.89099986	3.79892103
H	-1.02883802	0.00000000	3.79892103
H	0.51441901	0.89099986	3.79892103

***Cl_3B–NCMe* (*LB*) (*C_1*)**

E = -1164.8

H = -1125.9

N_{imag} = 0

B	0.00591344	0.00000066	-2.15980040
Cl	0.88730615	-1.52357248	-2.18004484
Cl	-1.75066590	0.00000165	-2.26526338
Cl	0.88730709	1.52357345	-2.18003610
N	-0.07267637	-0.00000718	0.80411524
C	0.01061162	0.00000098	3.42192412
C	-0.03611180	-0.00000330	1.96266893
H	0.53691743	-0.89099283	3.78243799
H	-1.00551886	0.00000206	3.83210565

H	0.53691720	0.89099699	3.78243289

$Cl_3B–NH_3$ (C_{3v})

E = -802.2

H = -768.2

N_{imag} = 0

B	0.00000000	0.00000000	-0.83678344
Cl	0.89492620	-1.55005765	-1.31180914
Cl	-1.78985240	0.00000000	-1.31180914
Cl	0.89492620	1.55005765	-1.31180914
N	0.00000000	0.00000000	0.80478106
H	0.96305038	0.00000000	1.15596832
H	-0.48152519	-0.83402610	1.15596832
H	-0.48152519	0.83402610	1.15596832

$Cl_3B–NH_3$ (C_1)

E = -802.2

H = -768.2

N_{imag} = 0

B	-0.00015696	-0.00000006	-0.83677797
Cl	0.89495682	-1.54998867	-1.31165537
Cl	-1.78993599	0.00000011	-1.31209996
Cl	0.89495720	1.54998848	-1.31165503
N	-0.00004351	-0.00000001	0.80477150
H	0.96310173	0.00000038	1.15567841
H	-0.48143937	-0.83403701	1.15610692
H	-0.48143994	0.83403677	1.15610668

$Br_3B–N_2$ (SB) (C_{3v})

E = -655.4

H = -641.9

N_{imag} = 0

B	0.00000000	0.00000000	-0.65103459
Br	0.97473634	-1.68829287	-1.06867449
Br	-1.94947269	0.00000000	-1.06867449
Br	0.97473634	1.68829287	-1.06867449
N	0.00000000	0.00000000	1.03876729
N	0.00000000	0.00000000	2.14579370

$Br_3B–N_2$ (SB) (C_1)

E = -655.4

H = -641.9

N_{imag} = 0

B	0.00028045	0.00000011	-0.65082176

Br	0.97504859	-1.68813358	-1.06912923
Br	-1.94947114	0.00000018	-1.06761844
Br	0.97504843	1.68813366	-1.06912986
N	0.00011489	0.00000001	1.03858534
N	-0.00102121	-0.00000038	2.14561688

***Br_3B–N_2* (*TS*) (*C_{3v}*)**

E = -655.4

H = -642.5

$\boldsymbol{N_{imag}}$ = 1, ν = -139.3264 cm^{-1}

B	-0.00000000	0.00000000	-0.55361993
Br	-0.97417800	-1.68732579	-0.96047858
Br	-0.97417800	1.68732579	-0.96047858
Br	1.94835600	-0.00000000	-0.96047858
N	-0.00000000	0.00000000	1.16421922
N	0.00000000	-0.00000000	2.27083643

***Br_3B–N_2* (*TS*) (*C_1*)**

E = -655.4

H = -642.5

$\boldsymbol{N_{imag}}$ = 1, ν = -152.3804 cm^{-1}

B	0.00024244	0.00019288	-0.13451397
Br	0.97419810	-1.68673905	-0.54002656
Br	-1.94806384	0.00006353	-0.53813483
Br	0.97420539	1.68690038	-0.54086490
N	-0.00019610	0.00017999	1.58726009
N	-0.00038599	-0.00059773	2.69378310

***Br_3B–N_2* (*LB*) (*C_{3v}*)**

E = -669.7

H = -657.3

$\boldsymbol{N_{imag}}$ = 0

B	0.00000000	0.00000000	-0.84107408
Br	0.96079882	-1.66415238	-0.85064356
Br	-1.92159765	0.00000000	-0.85064356
Br	0.96079882	1.66415238	-0.85064356
N	0.00000000	0.00000000	2.40898227
N	0.00000000	0.00000000	3.51152542

***Br_3B–N_2* (*LB*) (*C_1*)**

E = -669.7

H = -656.1

$\boldsymbol{N_{imag}}$ = 0

B	0.02571634	-0.00000138	-0.83211627

Br	0.97965037	-1.66408505	-0.93397084
Br	-1.88874218	-0.00000507	-0.65222008
Br	0.97964533	1.66408621	-0.93395711
N	0.12210983	-0.00000720	2.43152659
N	-0.30439355	0.00001248	3.44824065

***Br_3B–NCMe* (*SB*) (*C_{3v}*)**

E = -1122.1

H = -1082.8

N_{imag} = 0

B	0.00000000	0.00000000	-2.11922687
Br	0.97713441	-1.69244645	-2.66097659
Br	-1.95426882	0.00000000	-2.66097659
Br	0.97713441	1.69244645	-2.66097659
N	0.00000000	0.00000000	-0.57930048
C	0.00000000	0.00000000	2.01974531
C	0.00000000	0.00000000	0.57241173
H	0.51631983	-0.89429218	2.38874463
H	-1.03263966	0.00000000	2.38874463
H	0.51631983	0.89429218	2.38874463

***Br_3B–NCMe* (*SB*) (*C_1*)**

E = -1122.1

H = -1082.8

N_{imag} = 0

B	-0.00037558	-0.00000002	-2.11921897
Br	0.97727137	-1.69247465	-2.65993415
Br	-1.95412883	0.00000006	-2.66296544
Br	0.97727128	1.69247462	-2.65993419
N	-0.00121524	-0.00000001	-0.57930849
C	0.00024722	0.00000001	2.01972547
C	-0.00081574	0.00000003	0.57240128
H	0.51689880	-0.89428614	2.38829817
H	-1.03205208	-0.00000001	2.38957191
H	0.51689880	0.89428613	2.38829823

***Br_3B–NCMe* (*TS*) (*C_{3v}*)**

E = -1112.2

H = -1075.7

N_{imag} = 1, ν = -161.6613 cm^{-1}

B	-0.00000000	-0.00000000	2.55368554
Br	-0.96679885	1.67454473	2.80870201
Br	-0.96679885	-1.67454473	2.80870201
Br	1.93359770	0.00000000	2.80870201
N	0.00000000	0.00000000	0.29185494

C	-0.00000000	-0.00000000	-2.32121380
C	0.00000000	0.00000000	-0.86437015
H	-0.51495011	0.89191975	-2.69535418
H	-0.51495011	-0.89191975	-2.69535418
H	1.02990021	-0.00000000	-2.69535418

***Br_3B–NCMe* (*TS*) (*C_1*)**

E = -1112.2

H = -1075.7

N_{imag} = 1, ν = -166.3459 cm^{-1}

B	-0.00204634	-0.00021158	-1.68114839
Br	0.96292236	-1.67896513	-1.91855970
Br	-1.92960465	0.00842491	-1.97857086
Br	0.97733219	1.67058949	-1.91596853
N	-0.03798512	-0.00138295	0.57379301
C	0.00705589	0.00038112	3.18643924
C	-0.01659480	0.00007252	1.72979001
H	0.54136685	-0.88374692	3.55191483
H	-1.01660064	-0.01541093	3.57699675
H	0.51415427	0.90024948	3.55224745

***Br_3B–NCMe* (*LB*) (*C_{3v}*)**

E = -1113.9

H = -1076.7

N_{imag} = 0

B	0.00000000	0.00000000	-2.22615903
Br	0.96154077	-1.66543746	-2.27157349
Br	-1.92308153	0.00000000	-2.27157349
Br	0.96154077	1.66543746	-2.27157349
N	0.00000000	0.00000000	0.82467813
C	0.00000000	0.00000000	3.44409665
C	0.00000000	0.00000000	1.98399958
H	0.51436942	-0.89091398	3.82167965
H	-1.02873885	0.00000000	3.82167965
H	0.51436942	0.89091398	3.82167965

***Br_3B–NCMe* (*LB*) (*C_1*)**

E = -1115.1

H = -1076.2

N_{imag} = 0

B	0.06979346	0.30775198	-2.65332367
Br	1.72826514	-0.54649551	-2.16710317
Br	-1.14661287	-0.55475522	-3.84694830
Br	-0.32366101	2.06482495	-1.96397053
N	-1.55956439	-1.17418629	-0.07624465

C	0.29295201	0.01030496	1.34700053
C	-0.74273004	-0.65495982	0.56357116
H	-0.08726652	0.26412626	2.34263803
H	0.60505550	0.92885989	0.83777127
H	1.16376385	-0.64548226	1.45398590

***Br_3B–NH_3* (*C_{3v}*)**
E = -755.2
H = -721.7
N_{imag} = 0

B	0.00000000	0.00000000	-0.82249593
Br	0.97746302	-1.69301561	-1.34816023
Br	-1.95492604	0.00000000	-1.34816023
Br	0.97746302	1.69301561	-1.34816023
N	0.00000000	0.00000000	0.79948606
H	0.96381602	0.00000000	1.15140772
H	-0.48190801	-0.83468916	1.15140772
H	-0.48190801	0.83468916	1.15140772

***Br_3B–NH_3* (*C_1*)**
E = -755.2
H = -721.7
N_{imag} = 0

B	-0.00012060	0.00000001	-0.82249089
Br	0.97744000	-1.69297793	-1.34799298
Br	-1.95503978	0.00000099	-1.34846347
Br	0.97744138	1.69297706	-1.34799317
N	0.00000653	-0.00000001	0.79947590
H	0.96388407	0.00000169	1.15121431
H	-0.48180424	-0.83470181	1.15149147
H	-0.48180738	0.83470001	1.15149143

***I_3B–N_2* (*SB*) (*C_{3v}*)**
E = -607.2
H = -593.7
N_{imag} = 0

B	0.00000000	0.00000000	-0.57350641
I	1.09033148	-1.88850952	-1.10225332
I	-2.18066296	0.00000000	-1.10225332
I	1.09033148	1.88850952	-1.10225332
N	0.00000000	0.00000000	0.97766132
N	0.00000000	0.00000000	2.09137717

***I_3B–N_2* (*SB*) (*C_1*)**
E = -607.2

H = -593.7

$\boldsymbol{N_{imag}}$ = 0

B	0.00032122	-0.00000001	-0.57349985
I	1.09038752	-1.88855255	-1.10252484
I	-2.18050566	-0.00000002	-1.10172222
I	1.09038754	1.88855254	-1.10252476
N	0.00001832	0.00000000	0.97766411
N	-0.00060894	0.00000004	2.09137969

I_3B–N_2 (TS) (C_{3v})

E = -604.8

H = -592.2

$\boldsymbol{N_{imag}}$ = 1, ν = -301.7345 cm^{-1}

B	0.00000000	0.00000000	-0.52376176
I	-1.08448217	-1.87837822	-0.91825972
I	-1.08448217	1.87837822	-0.91825972
I	2.16896434	0.00000000	-0.91825972
N	0.00000000	0.00000000	1.35209919
N	-0.00000000	-0.00000000	2.45892049

I_3B–N_2 (TS) (C_1)

E = -604.8

H = -592.2

$\boldsymbol{N_{imag}}$ = 1, ν = -300.9181 cm^{-1}

B	0.00005292	-0.00001778	-0.21527667
I	1.08487020	-1.87912700	-0.60862753
I	-2.16974000	-0.00000723	-0.60855115
I	1.08486735	1.87911120	-0.60855194
N	-0.00000190	-0.00001006	1.66148396
N	-0.00004857	0.00005088	2.76829547

I_3B–N_2 (LB) (C_{3v})

E = -618.1

H = -606.0

$\boldsymbol{N_{imag}}$ = 2; ν = -10.00998i cm^{-1}

B	0.00000000	0.00000000	-0.90307639
I	1.07204001	-1.85682776	-0.90582499
I	-2.14408002	0.00000000	-0.90582499
I	1.07204001	1.85682776	-0.90582499
N	0.00000000	0.00000000	2.45332718
N	0.00000000	0.00000000	3.55599632

I_3B–N_2 (LB) (C_1)

E = -618.1

H = -604.8

$\boldsymbol{N_{imag}}$ = 0

B	0.03518267	0.00531129	-0.88997053
I	1.09766632	-1.85354907	-0.99364730
I	-2.09682345	0.00736942	-0.64780541
I	1.09783464	1.86074064	-1.03439457
N	0.15697534	-0.01460822	2.50397533
N	-0.39780349	-0.12396087	3.45061687

I_3B–NCMe (SB) (C_{3v})

E = -1073.9

H = -1034.9

$\boldsymbol{N_{imag}}$ = 0

B	0.00000000	0.00000000	-2.09778986
I	1.08992430	-1.88780427	-2.70863403
I	-2.17984861	0.00000000	-2.70863403
I	1.08992430	1.88780427	-2.70863403
N	0.00000000	0.00000000	-0.58685618
C	0.00000000	0.00000000	2.01339307
C	0.00000000	0.00000000	0.56731220
H	0.51630091	-0.89425941	2.38340290
H	-1.03260182	0.00000000	2.38340290
H	0.51630091	0.89425941	2.38340290

I_3B–NCMe (SB) (C_1)

E = -1073.9

H = -1034.9

$\boldsymbol{N_{imag}}$ = 0

B	0.00043842	-0.00000013	-2.09778928
I	1.08957624	-1.88796236	-2.70937048
I	-2.17993051	-0.00000001	-2.70705911
I	1.08957630	1.88796244	-2.70936949
N	0.00121724	-0.00000033	-0.58686470
C	-0.00019891	0.00000008	2.01337324
C	0.00099848	-0.00000020	0.56730127
H	0.51573511	-0.89425956	2.38389228
H	-1.03314750	0.00000017	2.38236019
H	0.51573510	0.89425989	2.38389191

I_3B–NCMe (TS) (C_{3v})

E = -1060.0

H =

$\boldsymbol{N_{imag}}$ = 1, ν = -170.3271 cm^{-1}

B	0.00000000	0.00000000	2.58471633
I	2.15792238	0.00000000	2.85331120

I	-1.07896118	1.86881560	2.85331120
I	-1.07896118	-1.86881560	2.85331120
N	0.00000000	0.00000000	0.26463532
C	-0.00000000	-0.00000000	-2.34873830
C	0.00000000	0.00000000	-0.89208334
H	1.02996401	-0.00000000	-2.72282121
H	-0.51498200	0.89197499	-2.72282121
H	-0.51498200	-0.89197499	-2.72282121

***I_3B–NCMe* (*TS*) (*C_1*)**

E = -1060.0

H = -1023.2

N_{imag} = 1, ν = -178.0817 cm^{-1}

B	-0.00228270	0.00006450	-1.72209512
I	1.08330364	-1.86816650	-1.97801703
I	-2.15495682	-0.00085204	-2.03387149
I	1.08120642	1.86993583	-1.97515087
N	-0.03578223	-0.00181628	0.58309550
C	0.00669358	0.00020449	3.19622013
C	-0.01774380	-0.00107620	1.73973784
H	0.52667671	-0.89228686	3.56211661
H	-1.01664971	0.00226440	3.58791822
H	0.52953488	0.89172866	3.56041204

***I_3B–NCMe* (*LB*) (*C_{3v}*)**

E = -1062.0

H = -1025.3

N_{imag} = 2; ν = -10.21047i cm^{-1}

B	0.00000000	0.00000000	-2.30191545
I	1.07256318	-1.85773393	-2.33697843
I	-2.14512637	0.00000000	-2.33697843
I	1.07256318	1.85773393	-2.33697843
N	0.00000000	0.00000000	0.84378608
C	0.00000000	0.00000000	3.46331969
C	0.00000000	0.00000000	2.00326882
H	0.51436588	-0.89090785	3.84094733
H	-1.02873177	0.00000000	3.84094733
H	0.51436588	0.89090785	3.84094733

***I_3B–NCMe* (*LB*) (*C_1*)**

E = -1063.9

H = -1025.3

N_{imag} = 0

B	-0.57344076	-0.40815941	-1.35622238
I	0.81488608	-1.37654763	-2.65040800

I	-1.96074337	-1.56495938	-0.18800826
I	-0.62573373	1.73878386	-1.23121734
N	1.88364680	-0.60915158	1.08958571
C	0.04632644	0.47161396	2.61161755
C	1.07514099	-0.13231830	1.77157714
H	-0.45811892	-0.29950258	3.20375260
H	-0.69561936	0.97322666	1.98017362
H	0.49365578	1.20701412	3.28951514

I_3B–NH_3 (C_{3v})
E = -704.8
H = -671.6
N_{imag} = 0

B	0.00000000	0.00000000	-0.81872115
I	1.08930658	-1.88673435	-1.39920404
I	-2.17861316	0.00000000	-1.39920404
I	1.08930658	1.88673435	-1.39920404
N	0.00000000	0.00000000	0.79850116
H	0.96394202	0.00000000	1.15349695
H	-0.48197101	-0.83479827	1.15349695
H	-0.48197101	0.83479827	1.15349695

I_3B–NH_3 (C_1)
E = -704.8
H = -671.6
N_{imag} = 0

B	0.00032770	0.00000009	-0.81871905
I	1.08930547	-1.88679237	-1.39946080
I	-2.17853149	0.00000045	-1.39865240
I	1.08930596	1.88679210	-1.39946126
N	0.00006895	-0.00000000	0.79849077
H	0.96380588	0.00000064	1.15400671
H	-0.48214057	-0.83477061	1.15322723
H	-0.48214191	0.83476970	1.15322753

F_3Al–NH_3 (C_{3v})
E = -947.5
H = -914.4
N_{imag} = 1, ν = -28.8932i cm^{-1}

Al	0.00000000	0.00000000	1.03038368
F	-1.64958089	-0.00000000	1.33475434
F	0.82479045	1.42857896	1.33475434
F	0.82479045	-1.42857896	1.33475434
N	0.00000000	0.00000000	-0.98701766
H	0.95340564	-0.00000000	-1.36040723

H	-0.47670281	-0.82567350	-1.36040723
H	-0.47670281	0.82567350	-1.36040723

***F_3Al–NH_3* (*C_1*)**
E = -947.6
H = -913.9
N_{imag} = 0

Al	-0.00000000	0.00000000	1.02782607
F	-1.64947702	-0.01518590	1.33592646
F	0.81158714	1.43608195	1.33592646
F	0.83788988	-1.42089606	1.33592646
N	-0.00000000	0.00000000	-0.99125667
H	0.65330859	-0.69715887	-1.35931382
H	-0.93041158	-0.21720240	-1.35931382
H	0.27710300	0.91436127	-1.35931382

***F_3Al–NCMe* (*C_{3v}*)**
E = -1320.3
H = -1281.0
N_{imag} = 1, ν = -33.0859i cm^{-1}

Al	0.00000000	0.00000000	2.36772838
F	-1.64459174	-0.00000000	2.68297245
F	0.82229587	1.42425823	2.68297245
F	0.82229587	-1.42425823	2.68297245
N	0.00000000	0.00000000	0.37489829
C	0.00000000	0.00000000	-0.77699916
C	-0.00000000	-0.00000000	-2.22773851
H	1.03233959	-0.00000000	-2.59560211
H	-0.51616979	0.89403231	-2.59560211
H	-0.51616979	-0.89403231	-2.59560211

***F_3Al–NCMe* (*C_1*)**
E = -1320.4
H = -1280.4
N_{imag} = 0

Al	-0.00000000	-0.00000000	2.36760762
F	-1.64456833	-0.02400700	2.68196109
F	0.80149350	1.43624145	2.68196109
F	0.84307483	-1.41223446	2.68196109
N	0.00000000	0.00000000	0.37485717
C	0.00000000	0.00000000	-0.77704092
C	0.00000000	0.00000000	-2.22687639
H	0.67433180	-0.78184742	-2.59481025
H	0.33993383	0.97491218	-2.59481025
H	-1.01426563	-0.19306476	-2.59481025

***$F_3Al–N_2$* (*C_{3v}*)**
E = -856.9
H = -842.3
N_{imag} = 0

Al	-0.00000000	-0.00000000	0.82677726
F	-1.65223106	-0.00000000	0.99423395
F	0.82611553	1.43087407	0.99423395
F	0.82611553	-1.43087407	0.99423395
N	-0.00000000	-0.00000000	-1.35480366
N	0.00000000	0.00000000	-2.45467542

***$F_3Al–N_2$* (*C_1*)**
E = -856.9
H = -842.3
N_{imag} = 0

Al	-0.15475853	0.13234856	-0.80114179
F	-1.80781578	0.17536112	-0.64787353
F	0.56728576	-1.26117359	-1.34399315
F	0.68199360	1.56346944	-0.89952075
N	0.25369232	-0.21695614	1.31329448
N	0.45960263	-0.39304939	2.37923475

***$Cl_3Al–NH_3$* (*C_{3v}*)**
E = -789.7
H = -757.1
N_{imag} = 0

Al	0.00000000	0.00000000	0.99959100
Cl	-2.09250000	0.00000000	1.39732800
Cl	1.04625000	1.81215800	1.39732800
Cl	1.04625000	-1.81215800	1.39732800
N	0.00000000	0.00000000	-1.02094400
H	0.95496300	-0.00000000	-1.39021000
H	-0.47748100	-0.82702200	-1.39021000
H	-0.47748100	0.82702200	-1.39021000

***$Cl_3Al–NH_3$* (*C_1*)**
E = -789.7
H = -757.1
N_{imag} = 0

Al	-0.20025134	-0.86661513	0.46062159
Cl	-2.26150655	-0.60128941	0.92500653
Cl	1.16521647	-0.77281363	2.09203909
Cl	0.25797584	-2.25381675	-1.08874074

N	0.20429285	0.88410534	-0.46991795
H	1.18156415	0.92373940	-0.77319060
H	-0.38509662	1.00665036	-1.29829414
H	0.03780519	1.68003982	0.15247621

***Cl_3Al–NCMe* (*C_{3v}*)**
E = -1162.2
H = -1124.0
N_{imag} = 1, ν = -40.0204i cm^{-1}

Al	-0.00000000	-0.00000000	2.33402500
Cl	-2.08549400	0.00002200	2.74752300
Cl	1.04276600	1.80608000	2.74752300
Cl	1.04272800	-1.80610200	2.74752300
N	-0.00000000	-0.00000000	0.34875800
C	-0.00000000	-0.00000000	-0.80403200
C	-0.00000000	0.00000000	-2.25367400
H	1.03215700	0.00152700	-2.62254800
H	-0.51740100	0.89311100	-2.62254800
H	-0.51475600	-0.89463800	-2.62254800

***Cl_3Al–NCMe* (*C_1*)**
E = -1162.2
H = -1124.0
N_{imag} = 0

Al	0.00080400	0.00032000	2.33385100
Cl	-2.07753500	-0.18358600	2.74220700
Cl	0.88011100	1.89135500	2.74860700
Cl	1.19693800	-1.70748700	2.75149000
N	0.00364500	-0.00066500	0.34861400
C	0.00189200	-0.00037900	-0.80417100
C	-0.00092000	0.00004800	-2.25342500
H	0.87098100	-0.55114200	-2.62424800
H	0.03946300	1.03148600	-2.62220900
H	-0.91538100	-0.47995500	-2.62070800

***Cl_3Al–N_2* (*C_{3v}*)**
E = -698.7
H = -685.4
N_{imag} = 0

Al	0.00000000	0.00000000	0.81098269
Cl	-2.09084944	0.00000000	1.03830277
Cl	1.04542472	1.81072873	1.03830277
Cl	1.04542472	-1.81072873	1.03830277
N	0.00000000	0.00000000	-1.41219330
N	-0.00000000	-0.00000000	-2.51369769

***Cl_3Al–N_2* (*C_1*)**

E = -698.7

H = -685.4

N_{imag} = 0

Al	-0.06575018	0.78062016	-0.20981511
Cl	-1.24535673	0.45738486	-1.92086251
Cl	1.99515833	1.12608146	-0.44845384
Cl	-1.00230607	1.41439720	1.56355050
N	0.11448496	-1.35922465	0.36533245
N	0.20376968	-2.41925902	0.65024852

***TriB–N_2* (*C_{3v}*)**

E = -5332.0

H = -5156.4

N_{imag} = 0

B	0.00000000	0.00000000	-1.27399074
C	2.75073517	0.00000000	-1.28462959
C	3.92233221	0.00000000	-0.50941196
H	4.74810582	0.00000000	1.48433718
C	-0.75172546	1.30202670	-0.66263028
C	-1.29335394	2.24015474	1.51703081
C	-1.92006282	3.32564636	0.88526219
C	-0.71271819	1.23446412	0.74710968
H	-2.44732175	4.23888562	-0.99753806
H	-1.41636842	2.45322207	-2.37154501
C	-0.75172546	-1.30202670	-0.66263028
H	0.00000000	0.00000000	2.40958353
C	1.42543638	0.00000000	0.74710968
C	-0.71271819	-1.23446412	0.74710968
C	0.00000000	0.00000000	1.31599207
H	2.52052162	0.00000000	2.60403532
C	1.50345093	0.00000000	-0.66263028
H	-1.41636842	-2.45322207	-2.37154501
H	-2.44732175	-4.23888562	-0.99753806
C	-1.29335394	-2.24015474	1.51703081
C	-1.92006282	-3.32564636	0.88526219
C	-1.96116611	-3.39683934	-0.50941196
C	-1.37536759	-2.38220654	-1.28462959
H	-2.37405291	-4.11198026	1.48433718
H	-1.26026081	-2.18283575	2.60403532
H	2.83273684	0.00000000	-2.37154501
H	4.89464350	0.00000000	-0.99753806
C	2.58670788	0.00000000	1.51703081
C	3.84012564	0.00000000	0.88526219

C	-1.37536759	2.38220654	-1.28462959
C	-1.96116611	3.39683934	-0.50941196
H	-2.37405291	4.11198026	1.48433718
H	-1.26026081	2.18283575	2.60403532
N	0.00000000	0.00000000	-2.78795251
N	0.00000000	0.00000000	-3.89969315

TriB–N $_2$ **(** C_1 **)**

E = -5332.0

H = -5156.4

N_{imag} = 0

B	-0.18136610	-2.09576851	2.20931080
C	1.36584827	-3.41025328	4.06545950
C	2.66791375	-3.71736026	4.49443023
H	4.77912028	-3.46453350	4.12588767
C	-0.10581635	-2.60386820	0.66980933
C	1.38345668	-2.40726781	-1.24539018
C	0.50569539	-3.20167001	-1.99946404
C	1.07898788	-2.11040647	0.08143868
H	-1.34716177	-4.30792533	-2.00746102
H	-1.89355963	-3.78296518	0.35162860
C	-0.09881763	-0.47523501	2.17004184
H	2.85630682	-0.90198584	0.50164361
C	2.29160993	-2.11979972	2.23185678
C	1.08564440	-0.09227545	1.50386018
C	1.95446766	-1.25637862	1.00863108
H	4.44231593	-2.03972923	2.10781506
C	1.17321180	-2.61395591	2.93785553
H	-1.88079587	0.22866542	3.17831350
H	-1.32538242	2.62451476	2.87674179
C	1.39523823	1.25577844	1.33591815
C	0.52294000	2.23685717	1.83260330
C	-0.65034608	1.86278090	2.49237934
C	-0.96140886	0.50320811	2.66115829
H	0.76259452	3.28997797	1.70265719
H	2.31033549	1.54528871	0.82138354
H	0.51596095	-3.80178748	4.62448764
H	2.81547618	-4.33916095	5.37490876
C	3.58342442	-2.42363220	2.65635168
C	3.77147652	-3.22594087	3.79260525
C	-0.97387009	-3.39212111	-0.08371785
C	-0.66791435	-3.69223740	-1.42160715
H	0.74138091	-3.43486649	-3.03551496
H	2.29883544	-2.02399956	-1.69390550
N	-1.43010341	-2.58514301	2.91148453
N	-2.34752728	-2.94261773	3.42774282
